# COVID-19 Mobile Health Apps: An Overview of Mobile Applications in Indonesia

**DOI:** 10.3389/fpubh.2022.879695

**Published:** 2022-05-04

**Authors:** Sujarwoto Sujarwoto, Trisfa Augia, Hendery Dahlan, Rindi Ardika Melsalasa Sahputri, Holipah Holipah, Asri Maharani

**Affiliations:** ^1^Department of Public Administration, Brawijaya University, Malang, Indonesia; ^2^Department of Public Health, Andalas University, Padang, Indonesia; ^3^Department of Mechanical Engineering, Andalas University, Padang, Indonesia; ^4^Portsmouth Brawijaya Centre for Global Health, Population and Policy, Malang, Indonesia; ^5^Department of Public Health, Faculty of Medicine, Brawijaya University, Malang, Indonesia; ^6^Faculty of Biology, Medicine and Health, The University of Manchester, Manchester, United Kingdom

**Keywords:** COVID-19, pandemic, public healthcare, mHealth apps, Indonesia COVID-19, Indonesia

## Abstract

**Background:**

Mobile health applications (mHealth apps) have been widely used for various purposes for mitigating the COVID-19 pandemic, such as self-assessment, contact tracing, disseminating information, minimizing exposure, and reducing face-to-face health consultation. The objective of this study is to systematically review COVID-19 related mHealth apps and highlight gaps to inform the development of future mHealth initiatives in Indonesia.

**Methods:**

A systematic search strategy using a PRISMA flowchart was used to identify mHealth apps available in Google Play and Apple Play stores. We searched mHealth apps using certain specific terms related to COVID-19 outbreaks. The inclusion criteria were apps-based smartphone users related to COVID-19 using local language, free of cost, available in the Google Play and Apple Play Stores, and supported by the Indonesian government. We excluded games, apps on infectious diseases unrelated to COVID-19 specifically, and apps with non-Bahasa Indonesia (Indonesian language). The selected mHealth apps were assessed based on two measures: (1) the WHO guidelines on digital health intervention and (2) the four dimensions of the mHealth technology fit framework. In addition, user feedback from experienced and non-experienced users was conducted to evaluate four dimensions of the apps.

**Results:**

A total of 339 mHealth apps were generated from the initial search, remaining seven selected apps that met inclusion criteria. The results highlighted that mHealth apps reviewed had still not been widely used by the general public. The applications were purposed to disseminate information, conduct a self-risk assessment, provide an online community forum, and telemedicine or teleconsultation regarding COVID-19. Data services, including data storage, aggregation, and data exchange, are available in most apps. The rarest function found was contact tracing and assisting health management and health workers, such as the availability of testing facilities, reporting test results, and prescribing medication. The main issues reported were the lack of data security and data privacy protection, integration and infrastructures, usability, and usefulness.

**Conclusion:**

Our study highlighted the necessity to improve mHealth apps' functions related to assisting health workers and the function of digital contact tracing. An effort to increase public awareness regarding the use of mHealth is also necessary to streamline the function of this innovation. Policymakers must consider usefulness, usability, integration, and infrastructure issues to improve their mHealth function.

## Introduction

Digital technology innovations are known as an enabler of health systems against pandemics. During Ebola and Zika epidemics, mHealth apps have improved access to testing, public awareness, supporting health workers, and contact tracing (1, 2). mHealth apps have also been developed to identify infected areas and contact tracing during the 2003 SARS-CoV-1 outbreak in China (3). In the current novel coronavirus disease (COVID-19) pandemic, many countries have developed mHealth apps to identify prevalent symptoms and infected areas, self-assessment, contact tracing, disseminate information, and minimize exposure and reduce face-to-face interaction between patients and health workers (3, 4).

A considerable amount of literature has been published to examine COVID-19 mHealth (5–15). Most of these studies focused on the goals and approaches of developing the apps quality, and technology advances (5–9). Although there are studies focused on the analysis of the features and functionalities, their evaluation is restricted to the general features of the apps such as usability and ease of use but did not include COVID-19 specific functionalities and features (10, 11). Some of them only discuss the breadth of common mHealth apps and their primary function during COVID-19 (3, 12). mHealth apps used in various countries during the pandemic were classified by the type of technology, targeted users, and function based on patient' needs (13). In addition, the review specifically related to the COVID-19 mHealth apps focused on specific functions, such as contact tracing (15), and only focused on specific populations, such as older people (14). Although prior studies have shown the utility and potential benefits of mHealth apps in preventing the pandemic, translating these ideas and early research into clinical tools on patients' mobile devices have received less attention (14).

Recent evaluation of mHealth apps concerning COVID-19 reported higher adoption of contact tracing systems is essential to lower the number of infections (16). Therefore, the success of a COVID-19 mHealth app depends on the adoption of the population. Nevertheless, low uptake rates were experienced in many countries (16). Many COVID-19 mHealth apps initiatives have not been as successful as originally expected in many countries. In a best-case scenario, Xia and Lee (17) posit that 90–95% of the population must use a contact tracing app to stop the spread of COVID-19 and allow normalcy without physical distancing. However, since March 2019, the apps have only been installed by about 9.3% of people in the 13 most populous countries with government-endorsed apps (18). Australia has reported the highest adoption rate with 21.6%, followed by Turkey with 17.3%, Germany with 14.4%, India with 12.5%, Italy with 7.2%, Peru with 6.8%, and Japan with 5%. The rest of the countries have an implementation rate below 5% (18). This evidence shows a need to understand the utilities and functionalities of COVID-19 mHealth apps and their gaps in a specific country to inform the development of future mHealth initiatives for improving apps uptakes.

Like many other countries, the Indonesian government has launched various mHealth apps for mitigating COVID-19. In April 2020, The Ministry of Communications and Informatics launched mHealth apps for COVID-19 screening called “*PeduliLindungi*”, while the Indonesian Social Security Administrator for Health (BPJS) launched their mHealth apps for COVID-19 screening called “Mobile JKN” (19). Some local governments and private organizations have also developed mHealth apps to mitigate the pandemic in their constituencies and organizations (19). Looking at the COVID-19 mitigation in Indonesia was crucial as it has the highest number of cases in the South-East Asia region and reached 1.51 million by 21 February 2022, with the number of fatalities reaching 146,202 deaths on the same date (20). Mitigating COVID-19 has thus become public health priority in Indonesia. With unexpected potential pandemics in the future, the objective of this study is to systematically review the utilities and functionalities of those mHealth apps and highlights their gaps to inform the development of future mHealth initiatives in the country.

## Methods

The mHealth apps reviewed were searched in the Google play store and Apple play store as Indonesians mainly use them. The search was conducted in the third week of August 2021 and updated on 7 November 2021. The inclusion and exclusion criteria were applied based on the PRISMA procedure to collect the data (digital applications) (21). The following inclusion criterion was used to choose the applications accessible in the mentioned stores: (1) apps launched for smartphone users and apps that are related to COVID-19 using *Bahasa Indonesia (Indonesian language)* or local language in Indonesia; (2) apps had to be free of cost and had to be launched and updated during the COVID-19 outbreak for the management of COVID-19 in Indonesia; (3) apps that available in Google Play Store and Apple Play Store, and (4) apps had to be launched and supported by the governments of Indonesia. We excluded games, apps on infectious diseases unrelated to COVID-19 specifically, and apps with non-*Bahasa Indonesia* (Indonesian language). We searched for the mHealth apps using the term “COVID-19,” “corona virus,” “epidemic,” and “pandemic” within the app title and description.

The functionalities of the COVID-19 apps were reviewed through the selected apps and the literature on epidemic management using digital-related programs (15, 22, 23). We categorized the mHealth apps' functionalities under the categories of the clients (general public), health workers, health system managers, and data services based on WHO recommendations on digital interventions for health system strengthening (24). We collected information about COVID-19 specific functions, the name, and the developer through selected apps then summarized the frequency and percentages of the information obtained from the selected apps. The detailed process of app reviews and results was available at https://figshare.com/s/bde8b7c1082234dd012e.

In addition to the systematic review, we conduct user feedback to understand users' evaluation of four dimensions of the apps: usefulness dimension, usability dimension, integration and infrastructure dimension, and other additional dimensions (25, 26). Each dimension consists of polar questions (a yes-no question) measuring the users' evaluation using the seven selected apps. Before field data collection, the instrument was translated into *Bahasa Indonesia* and had been verified by three academic experts in the field for approval. A pre-test of the survey platform was conducted for pilot testing. We asked five eligible participants to identify any vague or very complicated questions as well as response options. All of them reported that all questions and responses in the questionnaire were clear and easy to understand. The average time to finish all questions was 10–15 min. Validity and reliability tests were applied to the questionnaire. The validity coefficient (correlation coefficients) and the reliability coefficient (Cronbach's alpha) for each dimension were 0.81 and 0.81 for the usefulness dimension, 0.83 and 0.84 for the usability dimension, 0.86 and 0.86 for the integration and infrastructure dimension, and 0.82 and 0.82 for the others dimension. In addition, to estimate the reliability of the entire survey, the Spearman-Brown correction was applied. Kappa values were 0.83 indicates the instrument was statistically reliable.

Users were purposively selected based on their experience using the apps. We classified the users into two groups. Group 1 was users who had prior experience using all seven selected apps after meeting inclusion criteria (49 individuals), whole group 2 was users with no prior experience using those of the seven selected apps (49 individuals). All respondents were educated from high school or higher with IT and medicine background knowledge to ensure they were able to evaluate all of the app evaluation items. We used a non-probability sampling method based on convenience sampling to determine the number of samples in both groups (27). We followed Pett and Salkind who suggest *n* > 30 as the minimum sample size for using a parametric statistical test (28, 29). For group 2, we employed five facilitators to interview 49 participants. Before participants answered the questions, each facilitator asked them to install and use the apps. For group 1, we employed three facilitators to interview 31 users. Each facilitator recorded participant responses using the excel sheet form provided. An independent *t*-test was used to determine if there is a significant difference between the means of the groups.

This study received ethical approval from the Ministry of Education and Culture, University of Brawijaya (Number 123/KEP/UB/2021). Informed consent was obtained from all subjects involved in the study. Written informed consent has been obtained from the patient(s) to publish this paper.

## Results

### Systematic Review

**Figure 1:**
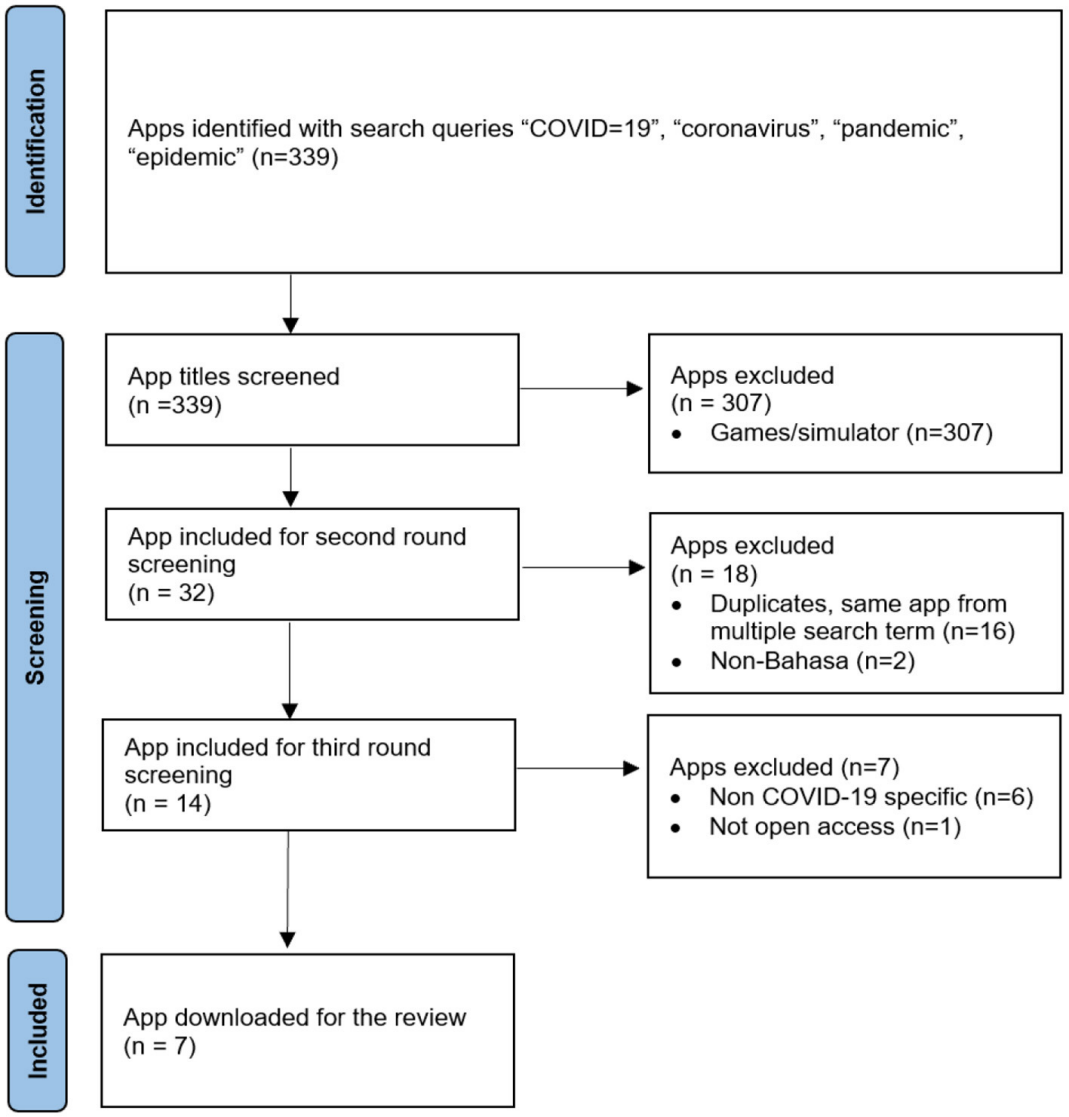
Screening process flowchart.

We identified 339 potential COVID-19 apps in Indonesia. Of these, 337 apps used the Indonesian language. Figure 1 provides the flowcharts of the apps selection procedure.

**Figure 2:**
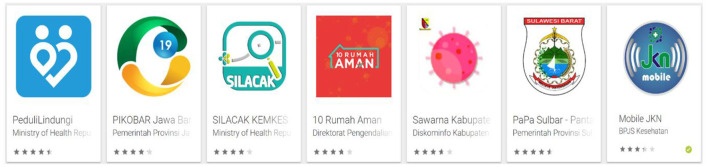
Selected COVID-19 related mHealth apps.

Of the 339 apps screened, 309 were excluded because they were games or simulators, eighteen apps were also excluded because of their duplication (*n* = 16) and using non-Bahasa (*n* = 2). We also excluded seven apps not specifically related to COVID-19 (*n* = 6) and non-free apps (in-app purchase). The remaining seven apps were analyzed in this study. The reviewed apps (*n* = 7) in Figure 2 consisted of four apps (57.1%) that were developed by the central government (*PeduliLindungi, 10 rumah aman, Mobile JKN*, and *SiLacak*), and three apps (42.8%) developed by the local government (*Pikobar Jabar, Sawarna Kabupaten Bandung*, and *Papa Sulbar*). By November 2021, the *PeduliLindungi* app was downloaded by 50 million people out of the 273.5 million Indonesian population (18.3% of the Indonesian population), while *Mobile JKN*, which belongs to the BPJS was downloaded by 10 million people (3.65% of the population) (30). Other apps developed by local governments were downloaded by fewer than fifty thousand individuals (1.7% of the total local government population).

Table 1 lists the function of the reviewed COVID-19 apps and their comparison with the WHO recommendation for digital health intervention. Of the seven reviewed apps, six (85.7%) apps (i.e., *PeduliLindungi, 10 Rumah, Mobile JKN, PIKOBAR, Sawarna*, and *Papa Sulbar*) provided a self-risk assessment function that screened users with a set of questions related to their symptoms, occupations, travel history, and contact history. Six (85.7%) apps (i.e., *PeduliLindungi, 10 Rumah, Mobile JKN, PIKOBAR, Sawarna*, and SiLacak) also provided information through chatbots or helplines. Most apps (71.4%) were developed to supply information dissemination regarding preventative measures (i.e., *PeduliLindungi, 10 Rumah, Mobile JKN, PIKOBAR*, and *Papa Sulbar*). Five apps (71.4%) offered online community forums for patients and family members and provided symptom trackers for the users (i.e., *PeduliLindungi, 10 Rumah, Mobile JKN, PIKOBAR*, and *Papa Sulbar*). The function of enrolment to health service and teleconsultation or testing appointments were available in five (71.4%) apps (i.e., *PeduliLindungi, 10 Rumah, Mobile JKN, PIKOBAR*, and *Papa Sulbar*). The reported COVID-19 test results and the prescription/medication management were only available in three (42.9%) apps (i.e., *PeduliLindungi, Mobile JKN*, and *PIKOBAR*). Only the *PeduliLindungi* app, which is sponsored by the Ministry of Information and Communication, offered specific facilities for the high-risk population, such as the availability of testing services and protective equipment. All apps still did not have facilities for client financial transactions. Most apps (85.7%) provided notifications for confirmed cases and deaths (i.e., *PeduliLindungi, 10 Rumah, Mobile JKN, PIKOBAR, Sawarna*, and *Papa Sulbar*), and four apps (57.1%) provided hotspot identification (i.e., *PeduliLindungi, Mobile JKN, PIKOBAR*, and *Sawarna*). However, only the *PeduliLindungi* app allowed contact tracing. As for data service management, six apps (85.7%) had provided data storage, aggregation, and visualization (i.e., *PeduliLindungi, 10 Rumah, Mobile JKN, PIKOBAR, Sawarna*, and *Papa Sulbar*). Five (71.4%) apps could record the location data or offer Bluetooth handshakes (i.e., *PeduliLindungi, 10 Rumah, Mobile JKN, PIKOBAR*, and *Sawarna)*. Finally, the location mapping of health facilities was available in four (57.1%) apps (i.e., *PeduliLindungi, Mobile JKN, Sawarna*, and *PIKOBAR*). The detailed information on seven selected apps based on the WHO guidelines on digital health intervention is available at https://figshare.com/s/bde8b7c1082234dd012e.

**Table 1:** Functionalities of COVID-19 mHealth apps and their comparison with WHO recommendations for digital health interventions (*n* = 7).

**WHO recommendations**	**COVID-19 related functions**	**Available**	**Not available**
		***n* (%)**	***n* (%)**
**Clients**
Targeted client communication	Availability of testing services and protective equipment for high-risk population	1 (14.3)	6 (85.7)
Untargeted client communication	Preventive measures and demystification	5 (71.4)	2 (28.6)
Client to client communication	Community forums for patients and family members	5 (71.4)	2 (28.6)
Personal health tracking	Symptom tracker	5 (71.4)	2 (28.6)
	Self-risk assessment	6 (85.7)	1 (14.3)
	Quarantine monitoring	4 (56.1)	3 (42.9)
Citizen based reporting	User feedback on services	3 (42.9)	4 (56.1)
On-demand information services to clients	Information provision through chatbots or helpline	6 (85.7)	1 (14.3)
Client financial transactions	Manage out of pocket payments by service users	0 (0)	7 (100)
**Health workers**
Client identification and registration	Enroll users for health services/clinical care	5 (71.4)	2 (28.6)
Client health records	Longitudinal tracking of user's health status	4 (56.1)	3 (42.9)
Health worker decision support	Job-aid for frontline health workers	0 (0)	7 (100%)
Telemedicine	Teleconsultation and testing appointments	5 (71.4)	2 (28.6)
Health worker communication	Provider to provider communication	0 (0)	7 (100)
Referral coordination	Manage referrals between points of service within the health sector	2 (28.6)	5 (71.4)
Health worker activity planning and scheduling	Electronic pass for the movement of the health workers during the lockdown	0 (0)	7 (100%)
Health worker training	Train new and existing healthcare staff	0 (0)	7 (100%)
Prescription and medication management		3 (42.9)	4 (56.1)
Laboratory and diagnostics imaging management	Testing for COVID-19	3 (42.9)	4 (56.1)
**Health system managers**
Human resource management	Human resource monitoring for hospital staff	0 (0)	7 (100)
	Participation/volunteer recruitment	2 (28.6)	5 (71.4)
Supply chain management	Monitor stock levels of health commodities	1 (14.3)	6 (85.7)
Public health event notification	Notification of confirmed cases	6 (85.7)	1 (14.3)
	Contact tracing	1 (14.3)	6 (85.7)
	Hotspot identification	4 (56.1)	3 (42.9)
Civil registration and vital statistic	Notification of deaths	6 (85.7)	1 (14.3)
Health financing	Accepting donations from contributors	2 (28.6)	5 (71.4)
Equipment and asset management	Monitor status of beds and ventilators	1 (14.3)	6 (85.7)
Facility management	Priority checklists for facility management	1 (14.3)	6 (85.7)
**Data services**
Data collection, management, and use	Data storage, aggregation, and visualization	6 (85.7)	1 (14.3)
	Prediction of future trends of disease	0 (0)	7 (100)
Location mapping	Map location of health facilities	4 (56.1)	3 (42.9)
	Location data recording or Bluetooth handshakes	5 (71.4)	2 (28.6)
Data exchange and interoperability	Data exchange across systems	5 (71.4)	2 (28.6)

### Users' Feedback

Users' feedback for seven selected apps was drawn from the questionnaire is presented in Table 2. Concerning usefulness dimension, participants report 21.4% of apps were able to consistently function from session to session (*p-*value = 0.91). Participants in both groups reported that 14.3% of apps work as advertised (*p-*value = 1.00), 14.3% of apps do not become clinically effective for the target population, disease, or disability (*p*-value = 1.00), and 14.3% of need more than 1 min to derive information they need (*p*-value = 1.00).

**Table 2:** Comparison of users' feedback for seven selected apps for group 1 and group 2.

**Usefulness dimension**	**Group 1**	**Group 2**	**Total**	***P*-value**
	**Yes (%)**	**Yes (%)**	**Mean Yes (%)**	
Will the app consistently function from session to session?	28.6	14.3	21.4	0.91
Does the app work as advertised?	14.3	14.3	14.3	1.00
Is the app clinically effective with demonstrated improved outcomes for the target population, disease, or disability?	14.3	14.3	14.3	1.00
What time is required for the user to derive some benefit from the app? Yes mean <1 min or vice versa	14.3	14.3	14.3	1.00
**Usability dimension**
Is the app pleasurable and enjoyable to use, or does it discourage repeat use?	28.6	28.6	28.6	1.00
Can the user easily-or with minimal training-use and understand the app?	14.3	14.3	14.3	1.00
Does the app work effectively with the user's culture (as defined by factors such as ethnicity and language)?	14.3	14.3	14.3	1.00
Does the app take into account socioeconomic status and the user's age, with potential implications for the user's digital health literacy?	14.3	14.3	14.3	1.00
Is the app usable by those with disabilities (e.g., incorporates screen readers for blind users, close captions for the hard-of-hearing and deaf communities)?	0.0	0.0	0.0	1.00
**Integration and infrastructure dimension**
Is the app containing personal health information?	100	100	100	1.00
Does the app share data with other apps, networks, and medical record systems?	100	100	100	1.00
Does the app work within its user's workflow?	14.3	14.3	14.3	1.00
Is the app anonymised?	0.0	0.0	0.0	1.00
Does the app contain a robust privacy policy addressing the type of information collected, rationale for collecting information, sharing of information, and user control?	0.0	0.0	0.0	1.00
Is the app's data encrypted on the device?	0.0	0.0	0.0	1.00
Is the app's data encrypted in transmission?	0.0	0.0	0.0	1.00
**Others**
Can the app provide information for either clinician education or point of care?	28.6	28.6	28.6	1.00
Does the app provide a differential diagnosis?	28.6	28.6	28.6	1.00
Can the app be used to educate or train patients, families, and/or support staff?	28.6	14.3	21.4	0.91
Can the app gather history (e.g., from the patients) and provide useful comprehensible output?	14.3	14.3	14.3	1.00

In terms of usability dimension, participants reported that 28.6% of apps are pleasurable and enjoyable to use (*p-value* = 1.00), 14.3% of apps can be used easily (*p-value* = 1.00), 14.3% of apps support the local language, and materials relevant to local culture and ethnicity (*p*-value = 1.00), 14.3% of apps take into account socioeconomic status and the user's age that support users with lack digital literacy (*p*-value = 1.00). All apps do not have tools that support disabled users (*p-*value = 1.00).

With regard to integration and infrastructure dimension, all users reported that all apps contain personal health information and share data with other apps, networks, and medical record systems (*p*-value = 1.00). Participants reported that 86% of the apps do not work within their user's workflow (*p-*value = 1.00). All users stated that the apps' data were not encrypted on the device, on transmission, were not anonymized, and did not contain a robust privacy policy to protect users (*p*-value = 1.00).

Furthermore, 28.6% of the apps could not be used to educate or train patients, families, and/or support staff, did not provide information for clinicians and point of care, and did not provide a differential diagnosis (*p*-value = 1.00). Only 21.4% of the apps were able to gather history of patients and provide useful information (*p*-value = 0.91), while 14.3% (*p*-value = 1.00) gave a comprehensive output.

## Discussion

This study aimed to evaluate COVID-19 related mobile apps used in Indonesia and highlight gaps to inform the development of mHealth related COVID-19 initiatives. We found very small investments from central and local governments in mHealth app development to deal with the pandemic crisis. Moreover, the proportion of the mHealth apps available for the population is relatively small, while evidence suggests that at least 70% of the population should have the apps installed for the digital contact tracing efforts to be effective (31). For example, *PeduliLindungi* and *Mobile JKN*, which national agencies developed, were downloaded by <20% of the national population. Prior studies have documented that inadequate Information and Communication Technology (ICT) infrastructure, low internet connectivity, low prescription, user resistance, and mHealth illiteracy are the main barriers to mHealth adoption in Indonesia, which is also commonly found in other developing countries (32–34).

All apps still did not meet the WHO recommendation for digital health information for COVID-19 mitigation (24). Most apps were used to disseminate COVID-related information on preventative strategies in which information provision was also delivered through chatbots or helplines. Despite that, a few apps are used to educate or train patients, families, and support staff. This finding corroborates a previous review of COVID-19 apps in East and South-East Asia and highlights the primary function of COVID-19 mHealth apps in most countries in the region for dissemination purposes (35). While interactive services and targeted client communication are crucial (11), most apps were still not designed for interactive engagement with users. For example, most of them have no user feedback on services and no facilities for client financial transactions. Only one app (*PeduliLindungi*) provides information regarding testing services and equipment for a high-risk population. The user feedback also reported that all apps did not incorporate facilities for disabled people and local language.

Most of the apps were not designed to assist health workers and health system managers. There is no function for health worker decision support, communication, activity planning, scheduling and training, hospital staff/human resources, monitoring, health commodity stock monitoring, and the movement system for health workers using electronic passes. Users also reported that they could not gather comprehensive output about patient history from the apps. These also confirm previous findings in a previous systematic review in East and South-East Asia (35). The review also found that the key feature to suppress coronavirus spread, contact tracing, was unavailable in most apps. Only one app reported contact tracing events. Most of the contact tracing activities have been manually conducted by surveillance officers, and therefore, the results of contact tracing can be directly reported for decision making (36).

Data and information privacy were the biggest issue in all apps. In the apps reviewed, when installing the apps and using the main features, users should input their data such as name, phone number, citizen registration number, email, Bluetooth interaction with other apps users, and real-time location. Data privacy concerns were also reported from user feedback. All users found that data encryption was not designed and anonymized, while the apps collect individuals' privacy preferences and personally identifiable information. The apps also did not include a robust privacy policy addressing personal and confidential information collected, the rationale for collecting information, sharing of information, and user control. These findings support evidence of previous mHealth related COVID-19 investigations in the country that data protection and security are a big concern as most of the apps have low-security protection technology (37, 38). The threat to privacy and personal data was also addressed in prior mHealth related COVID-19 evaluation in East and South-East Asia (11, 35, 39).

Issues of synchronization were also found in all apps. While most apps provided data exchange, storage, and aggregation, the apps did not integrate with each other. Each app had been developed with its own function, design, and platform. There is no data integration between central government apps and local government apps. The local government-initiated apps were designed only for people in their jurisdiction and cannot be synched to central government apps. With the characteristics of a fragmented, decentralized health care system in which the government system consists of many tiers of government organization, the current mHealth apps can be detrimental for technology-assisted COVID-19 contact tracing as the technology was unable to monitor the movement of people across jurisdictions. Previous studies suggest that single national contact tracing, which is incorporated with specific contacts information and the local health system, is preferable in such a fragmented decentralized health system (31).

Based on the research, there are several recommendations that mobile app developers can consider to improve their existing COVID-19 apps or create a high-quality COVID-19 mobile app in the future. First, the developers must implement the core data protection principles such as the General Data Protection Regulation (GDPR) to ensure that the app is secure and provide assurance to the users that all shared information is kept confidential. Second, creating an application integration network is essential to allow applications to communicate with each other so that work processes can be done more effectively and efficiently. For example, application integration between central and local governments would be very useful to maintain, manage, and keep the apps up to date while alleviating data duplication and redundancy across governments. A collaboration with local health authorities to develop a mHealth app can increase the reliability of the app, which will encourage more users to be engaged in its use. Third, improvement of user interface designs of existing apps is needed. For example, to increase the apps' uptake of the public, the apps should take into account socioeconomic status and the user's age, the local language, and ethnicity as well as those with disabilities. The apps should be made available without requiring any payment in both the Apple App Store and the Google Play Store to make them more accessible to the public. It is also crucial to categorize mobile apps into appropriate categories to enable users to find an app easily and thus improve its user uptake. Fourth, the findings suggested designing an app that can assist health workers and health system managers. For example, they would need to add functions for health worker decision support, contact tracing, notification of confirmed cases, monitoring status of beds and ventilators, priority checklists for facility management, and a telemedicine system. Adoption of mHealth and telemedicine in the current pandemic requires health workers to use videoconferencing, while the medical care system is still managing the outbreak (9, 40). Hence, the application of mHealth has become timely while providing great potential to protect health workers and patients.

## Conclusion

mHealth apps for COVID-19 in Indonesia are mainly designed for disseminating information, conducting a self-risk assessment, providing an online community forum, and telemedicine or teleconsultation regarding COVID-19. The least function found was contact tracing and assisting health management and health workers, such as availability of testing facilities, reporting test results, and prescribing medication. The main issues were data security and data privacy protection, integration and infrastructures, usability, and usefulness. This study suggests the necessity to improve the usefulness, usability, integration, and infrastructure of mHealth apps, especially data security and data privacy protection.

The study was limited by the fact that COVID-19 mHealth apps selected were limited to free applications available in the Google Play Store and Apple Play Store. We were unable to review in-app purchases. Another limitation is that we did not include web-based applications. We did not perform a more robust search in publication indexes such as PubMed, Web of Science, and Scopus. New COVID-19 mobile apps may be launched that could not be included in this review. Moreover, our sample was based on convenience sampling which was characterized by insufficient power to identify differences in population subgroups. The potential bias of the sampling technique because under-representation of subgroups in the sample in comparison to the population of interest may occur (41). Future research may address these limitations by including non-free apps and conducting apps review based on a database such as PubMed, Web of Science, and Scopus focusing on technologies, functions, and features of mHealth apps that can be used by medical practitioners, application developers, and governments to collaborate in the process of containing the spread of coronavirus. More importantly, future studies should use probability sampling methods based on a sample of the general population to get a more reliable statistical inference of the population regarding COVID-19 mHealth apps uptake.

## Data Availability Statement

The original contributions presented in the study are included in the article/Supplementary Material, further inquiries can be directed to the corresponding author/s.

## Author Contributions

SS, TA, HD, HH, and AM prepared study design. RS, HH, and TA collected data and conduct data analyses. SS and AM wrote the main manuscript text. All authors reviewed the manuscript. All authors contributed to the article and approved the submitted version.

## Funding

This study was supported by LPDP and ISF under the International Collaboration Productive-Innovative Research Funding Scheme Number. PRJ-71/LPDP/2021 dan No. 01/DIPI/2021 dated April 14, 2021.

## Conflict of Interest

The authors declare that the research was conducted in the absence of any commercial or financial relationships that could be construed as a potential conflict of interest.

## Publisher's Note

All claims expressed in this article are solely those of the authors and do not necessarily represent those of their affiliated organizations, or those of the publisher, the editors and the reviewers. Any product that may be evaluated in this article, or claim that may be made by its manufacturer, is not guaranteed or endorsed by the publisher.

## Supplementary Material

Supplementary Table 1 Search strategy.

Supplementary Material Functionalities of the coronavirus disease-19 apps.
