# Supplementary material for: COVID-19 Mobile Health Apps: An Overview of Mobile Applications in Indonesia
Source: Front Public Health. 2022 May 4;10:879695. doi: 10.3389/fpubh.2022.879695 (PMC9114306; doi:10.3389/fpubh.2022.879695)
Supplement: Supplementary Material — Functionalities of the coronavirus disease-19 apps. [file Data_Sheet_1.pdf]

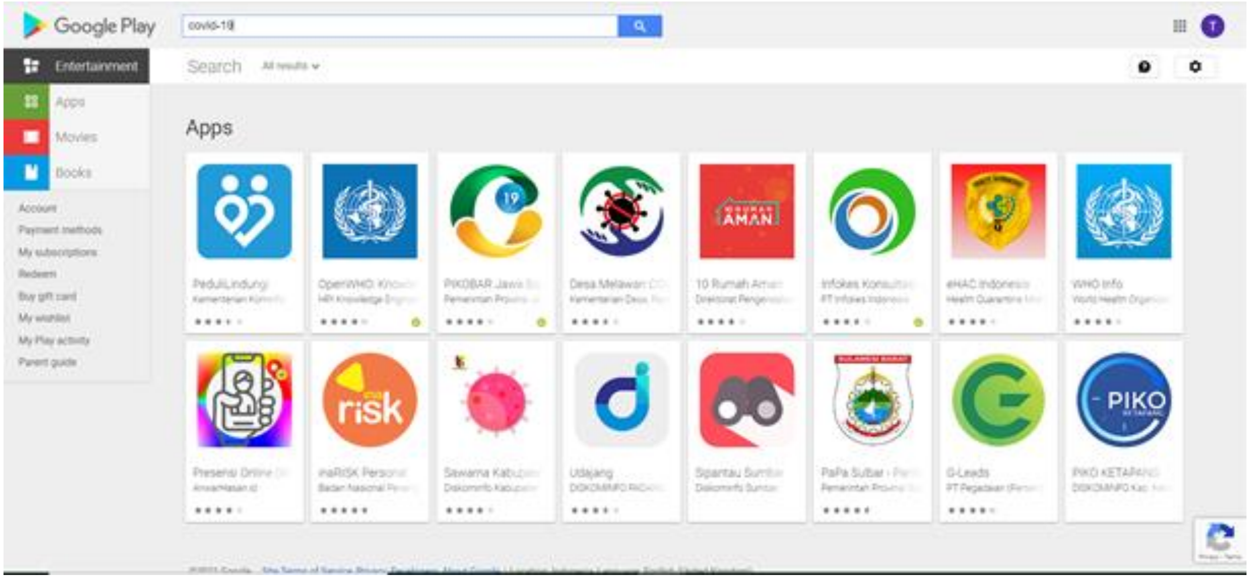

Keyword: COVID-19 (n= 16)

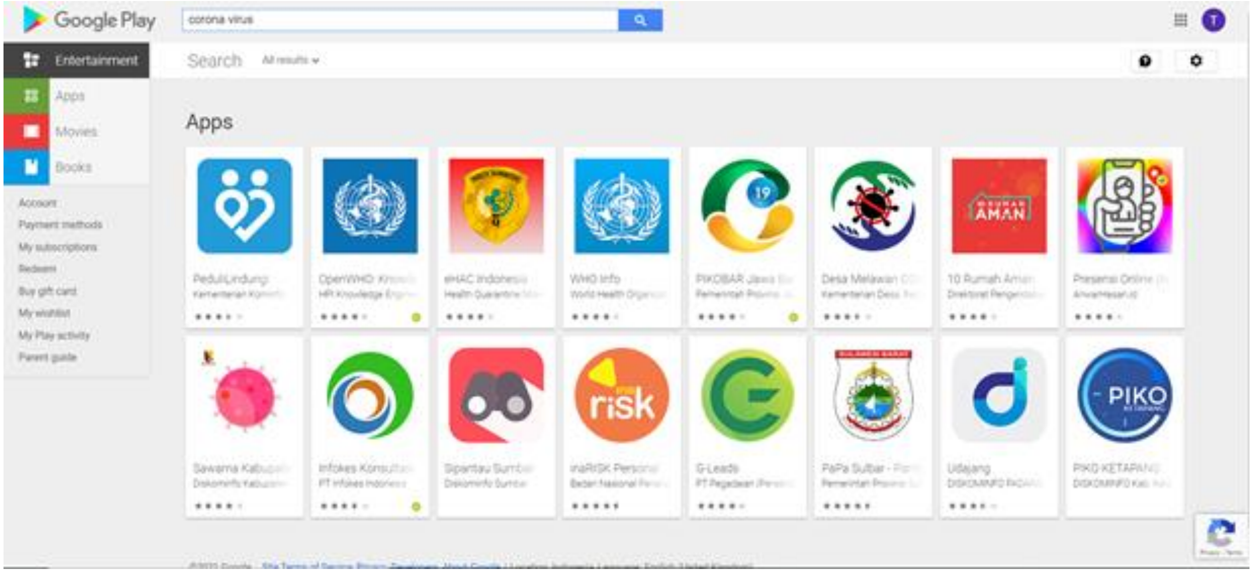

Keyword: corona virus (n=16)

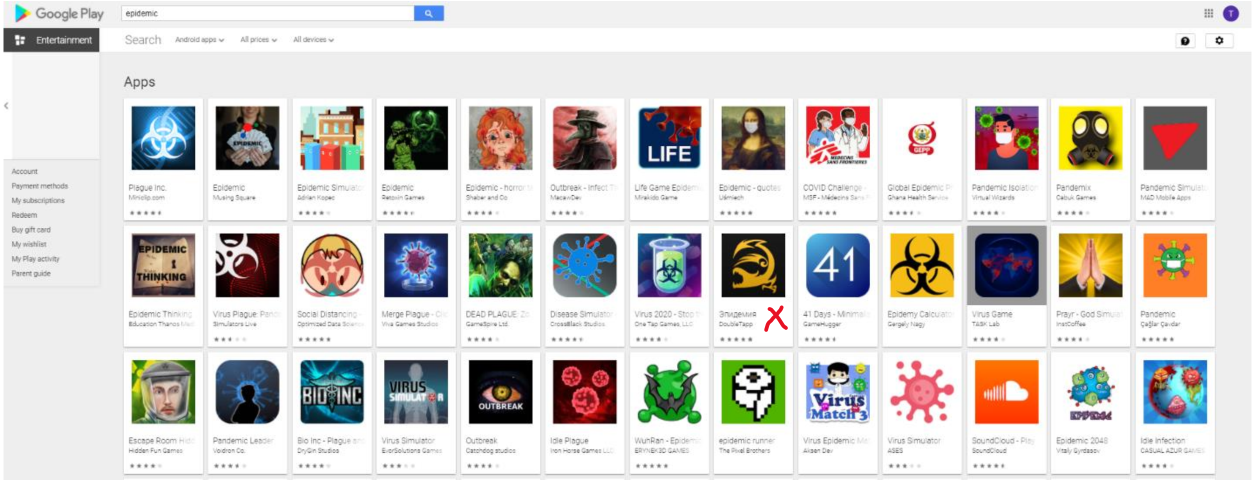
Google Play
epidemic
Entertainment
Search
Android apps
All prices
All devices
Apps
Account
Payment methods
My subscriptions
Redeem
Buy gift card
My wishlist
My Play activity
Parent guide

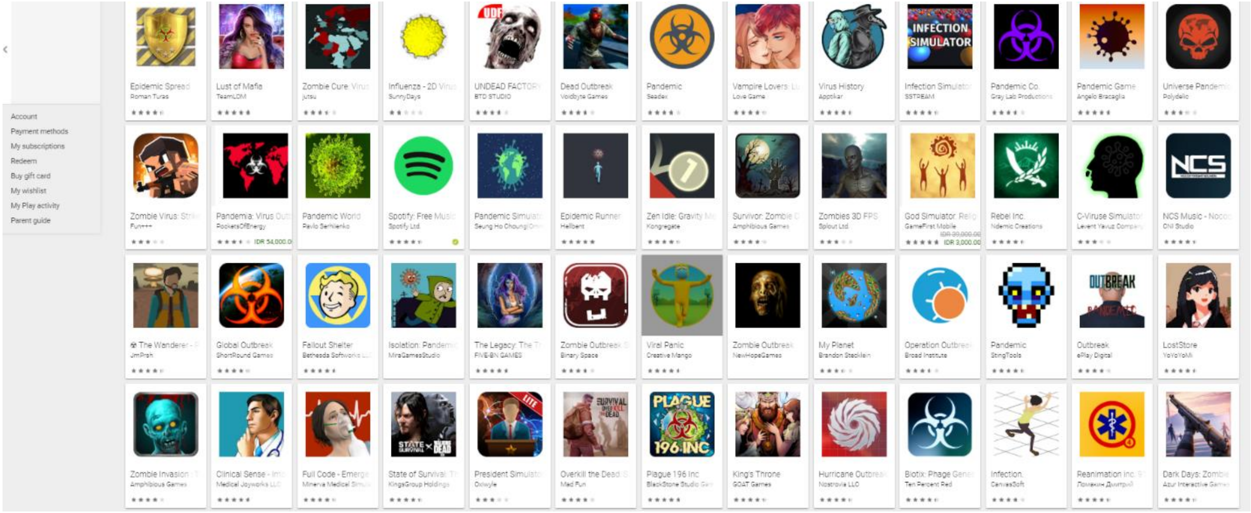
Account
Payment methods
My subscriptions
Redeem
Buy gift card
My wishlist
My Play activity
Parent guide

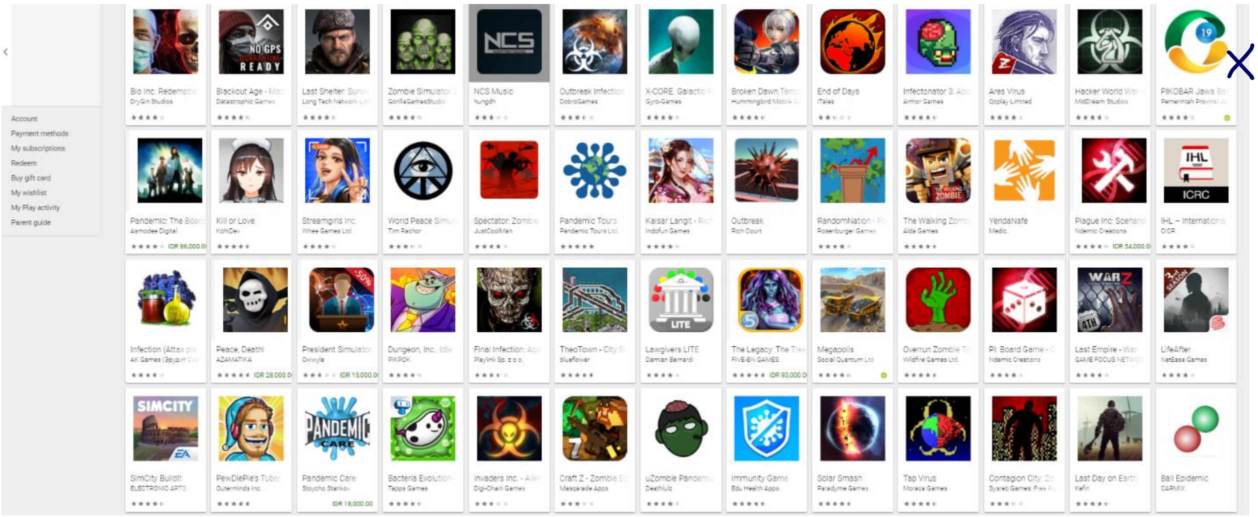
Account
Payment methods
My subscriptions
Redeem
Buy gift card
My wishlist
My Play activity
Parent guide

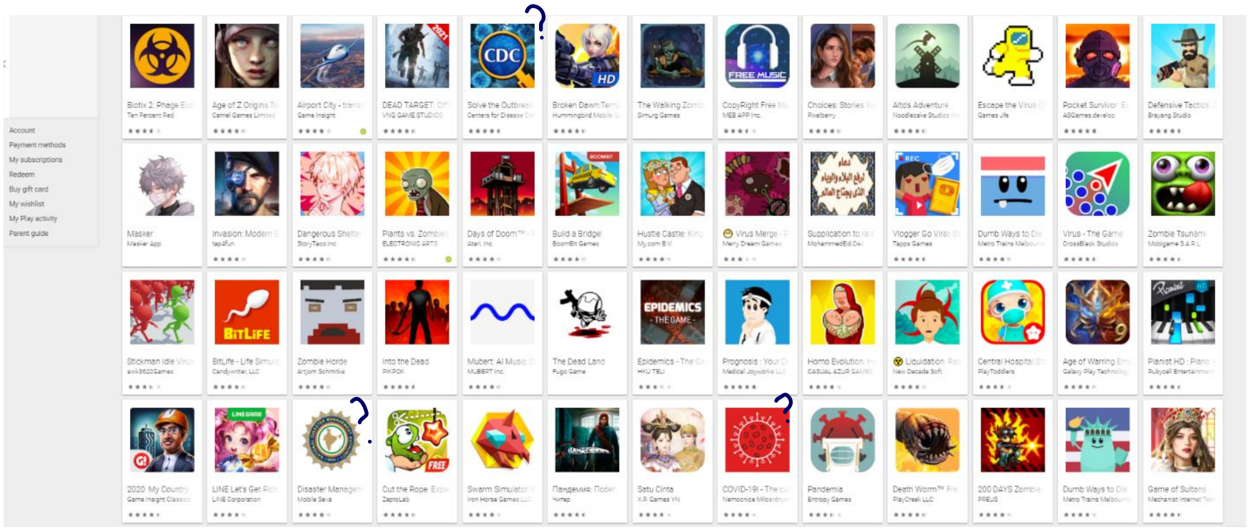

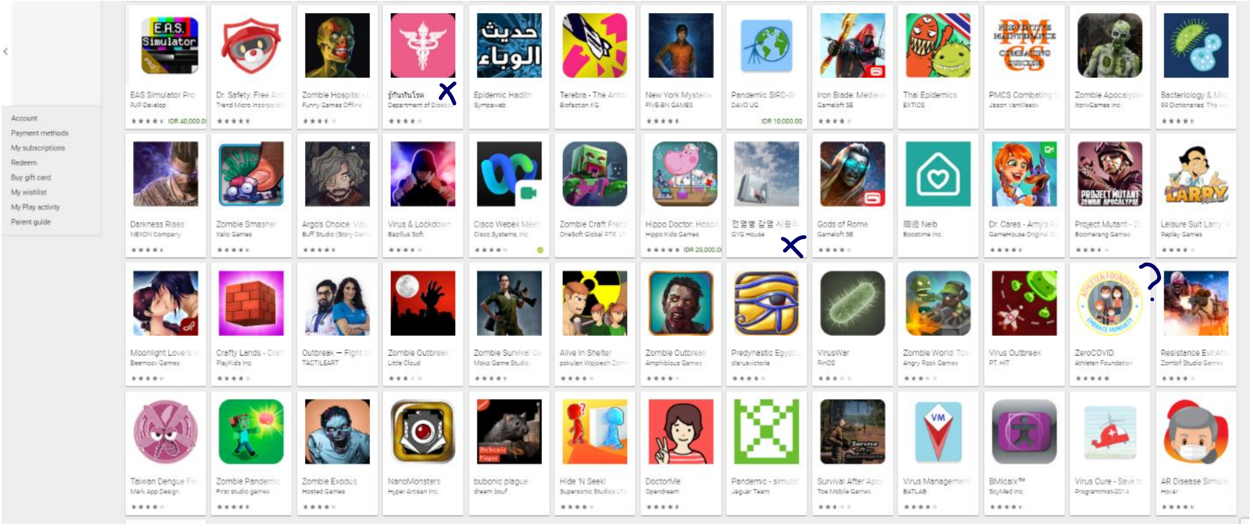

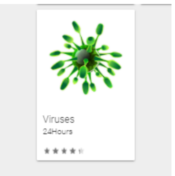

Keyword: epidemic (n=153)

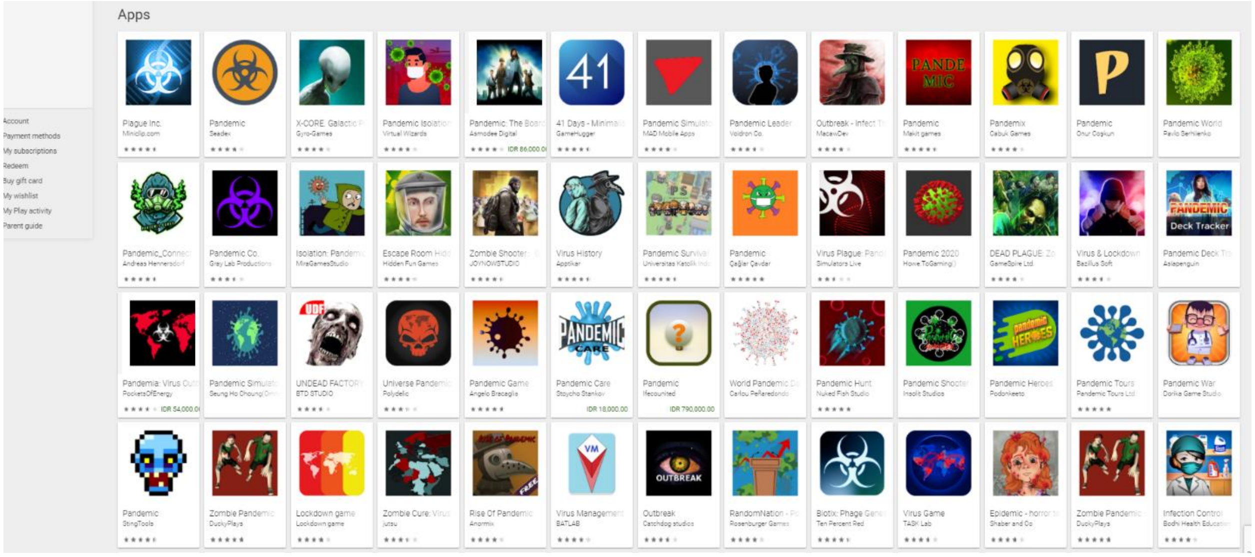
Apps
Account
Payment methods
My subscriptions
Redeem
Buy gift card
My wishlist
My Play activity
Parent guide

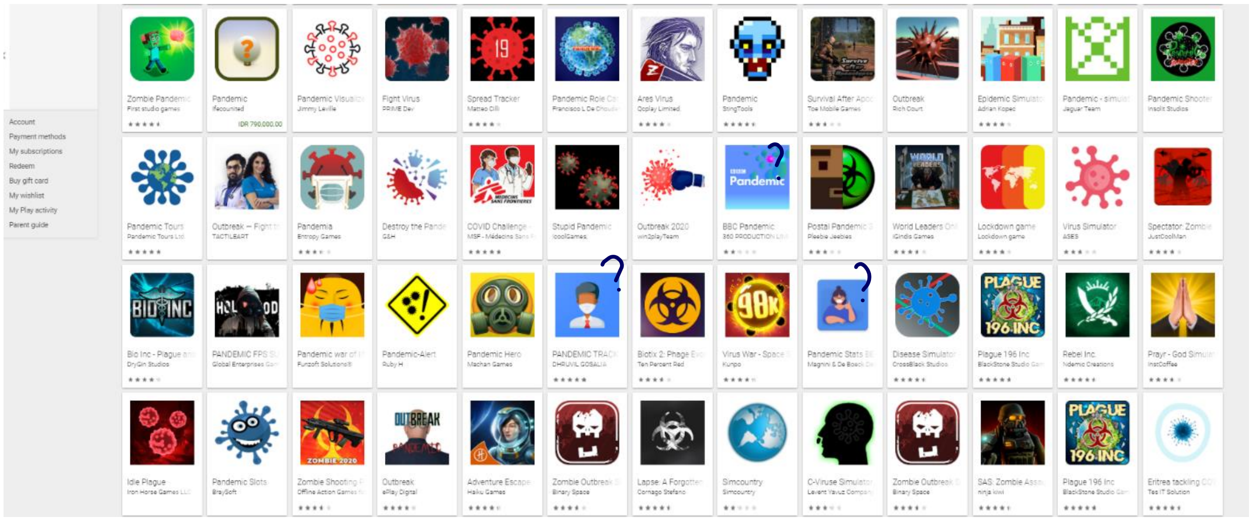
Account
Payment methods
My subscriptions
Redeem
Buy gift card
My wishlist
My Play activity
Parent guide

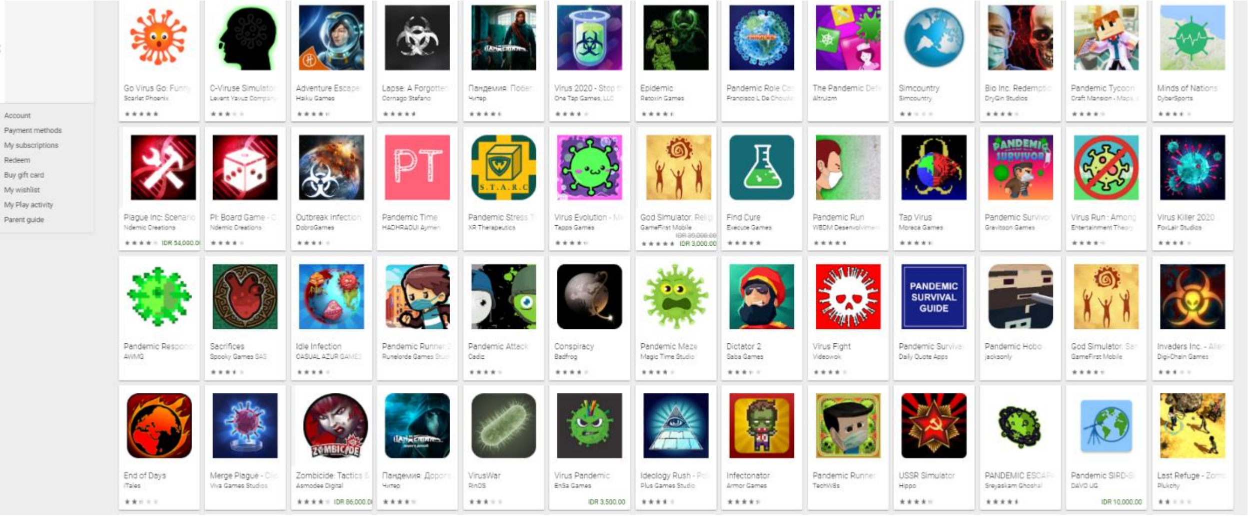
Account
Payment methods
My subscriptions
Redeem
Buy gift card
My wishlist
My Play activity
Parent guide

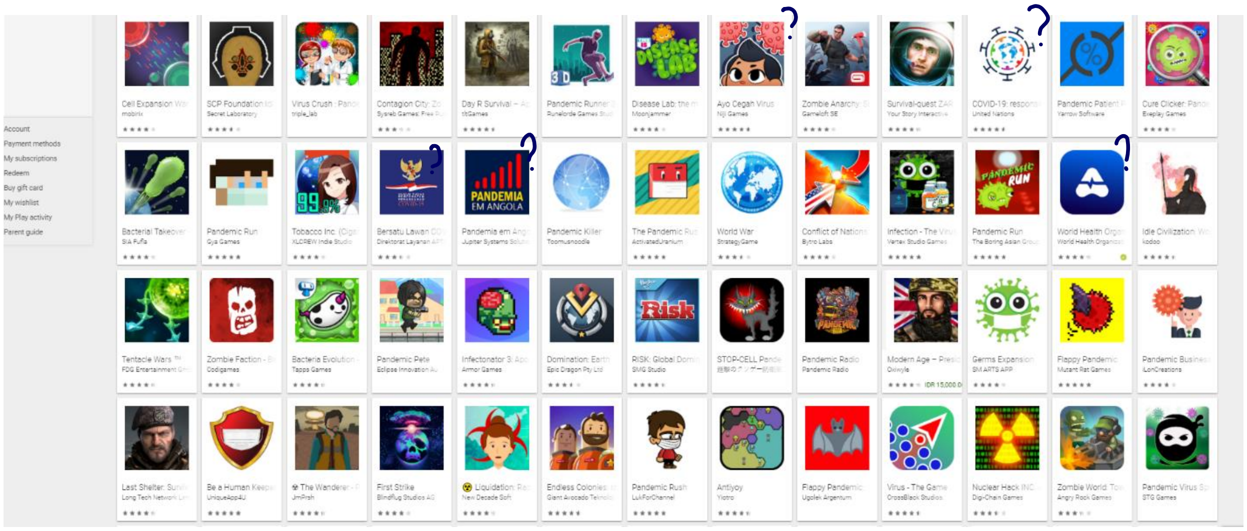

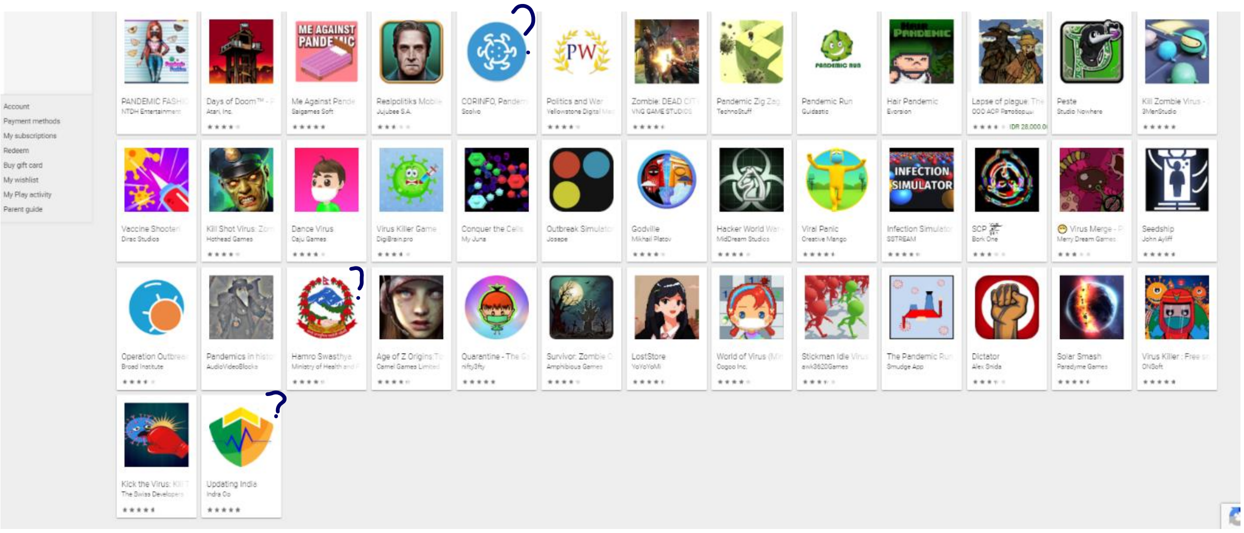

Keyword: pandemic (n=154)
